# Changing of the guards at the Scandinavian Journal of Work, Environment & Health

**DOI:** 10.5271/sjweh.4205

**Published:** 2025-01-01

**Authors:** Reiner Rugulies

With the first quarter of the 21st century in the books and the first issue of the year of the Journal in front of you, we would like to use this opportunity to wish all our readers, authors and reviewers a happy and healthy New Year. May your wishes, aspirations, and resolutions for 2025 come true.

One of the main challenges for the second quarter of the 21st century is the current and upcoming mass retirement of the baby boomer generation that is occurring in many countries, including and in particular in Europe (1). Numerous papers in the Journal have discussed the challenges and possible remedies (2–4). And now, the retirement of the boomers is also happening at SJWEH. As the New Year starts, we are saying goodbye to two Associate Editors as well as one Editor-in-Chief while saying hello to a new one.

The Associate Editors – Jens Peter Bonde and Mikko Härmä – are riding off into the sunset after decades-long service to the journal. Mikko has been with the Journal for more 30 years. He is a distinguished scholar in working time research and the effects of working time arrangements on workers’ health, which has evolved as a key topic in occupational health and SJWEH (5). Mikko joined the Journal as an Assistant-Editor-in-Chief in 1994 and became Editor-in-Chief in 2000, when the Journal’s inaugural Editor-in-Chief, Sven Hernberg, retired. For 20 years, Mikko was at the helm and steered the Journal towards its current position as one of the internationally leading occupational health journals. He recently reflected on this process in a presentation on the “changing business of running a scientific journal”, which is available on the Journal’s home page (6). At the end of 2019, Mikko stepped down as Editor-in-Chief, but remained on the Editorial Board as an Associate Editor, a position from which he is now retiring. Jens Peter has served as an Associate Editor since 2010. As an occupational physician and distinguished occupational epidemiologist, he had published extensively on a broad range of topics, including but not limited to reproductive health, cardiovascular disease, musculoskeletal disorders, mental disorders, and, most recently, COVID-19. For a research journal, it is stroke of luck to have a scholar with such a broad expertise on the Editorial Board, who can be assigned to a large proportion of the submitted papers. On a personal level, I would like to add that I am very grateful to Jens Peter for the many discussions we had on the methodological challenges in studying the health effects of psychosocial working conditions. These discussions influenced my research quite a bit.

The third retirement is that of Alex (Lex) Burdorf. Lex joined SJWEH as an Associate Editor in 2006 and became Co-Editor-in-Chief at the end of 2018, first together with Mikko Härmä and then with me. As an occupational hygienist and public health researcher, Lex is known for his seminal contributions to exposure assessment methods, musculoskeletal disorders, working-life expectancy and labor market participation, and causal inference debates. Sharing the Editor-in-Chief position with Lex for the past five years has been a privilege. It was educational, effective and, last but not least, a lot of fun. Fortunately for the Journal, Lex will stay on as an Associate Editor.

After saying all these goodbyes, we can also say one hello. Join me in welcoming Annina Ropponen, professor at the Finnish Institute of Occupational Health, who has been an Associate Editor at the Journal since 2018, as our new Co-Editor-in-Chief. Annina’s research covers a broad area in occupational health and includes, among other things, research on musculoskeletal disorders, working hours, shift work, remote work, sustainable employment, and sickness absence. I am very happy that Annina agreed to take on this new role and am very much looking forward to working together on the future for the Journal.

As we turn the page on this remarkable chapter and embrace the changes ahead, we remain deeply grateful for the invaluable contributions of our retiring editors and excited to welcome new leadership. With this blend of continuity and fresh perspectives, we are confident that the Journal will continue to thrive as a leading voice in occupational health research. Here’s to building on our legacy and shaping an even brighter future together—thank you for being part of this journey.
